# Real‐world emetic risk of chemotherapy and the corresponding antiemetic therapy in Japan: A study based on a nationwide database

**DOI:** 10.1002/cnr2.1482

**Published:** 2021-06-27

**Authors:** Ayako Okuyama, Narikazu Boku, Takahiro Higashi

**Affiliations:** ^1^ Center for Cancer Control and Information Services National Cancer Center Chuo‐ku Japan; ^2^ Division of Gastrointestinal Medical Oncology National Cancer Center Hospital Chuo‐ku Japan

**Keywords:** chemotherapy, nausea, neoplasms, psychological distress, registries, vomiting

## Abstract

**Background:**

Chemotherapy‐induced nausea and vomiting (CINV) is a major concern of patients with cancer, leading to suboptimal treatment.

**Aim:**

This study assessed the emetic risk associated with intravenous and oral chemotherapy and the prophylactic antiemetic drugs by cancer type in a real‐world setting.

**Methods and Results:**

We used the health services utilisation data for patients with cancer diagnosed in 2016. Patients aged at least 20 years at the time of diagnosis and who started their first course of chemotherapy were included. The emetic risk of chemotherapy was determined according to the cancer type and was classified based on clinical practice guidelines. The prescription of antiemetic drugs was assessed. Overall, 172 133 patients were evaluated, of whom 121 103 (70.4%) received intravenous chemotherapy. High‐emetic‐risk chemotherapy (HEC) was prescribed in 46 458 (27.0%) patients. HEC was prescribed most for patients with oesophageal cancer (80.3%), followed by malignant lymphoma (60.2%) and breast cancer (53.8%). Moderate‐emetic‐risk chemotherapy (MEC) was prescribed in 60 528 (35.2%) patients and was mostly prescribed for small cell lung cancer (59.9%). Meanwhile, more than 50% of the chemotherapy prescribed for patients with gastric, colorectal, and pancreatic cancer was low‐emetic‐risk chemotherapy. HEC was accompanied by three‐drug antiemetic prophylaxis in more than 90% of patients with small cell lung, non‐small cell lung, breast, and oesophageal cancer, whereas only 13.5% of patients with malignant lymphoma were administered CHOP (cyclophosphamide, doxorubicin, vincristine sulphate, and prednisolone) with prophylaxis.

**Conclusion:**

The risk of CINV differs with cancer type. HEC was less prescribed compared with MEC. Most patients received the recommended anti‐emetic prophylaxis.

## 1 INTRODUCTION

Chemotherapy‐induced nausea and vomiting (CINV) is a serious adverse event of chemotherapy.^1^, ^2^, ^3^ The frequency of CINV depends primarily on the emetic potential of the chemotherapeutic agent used. There are effective antiemetic agents for the prevention of CINV,^4^ which alleviate CINV significantly.^5^ Vomiting is observed in more than 90% of patients receiving high‐emetic‐risk chemotherapy (HEC) without prophylaxis^6^; this prevalence is reduced to approximately 30% when antiemetics are administered.^6^, ^7^, ^8^ Several guidelines of antiemetic therapy for chemotherapy recommend prescriptions based on the emetic risk of the chemotherapeutic agent used.^9^, ^10^

In clinical practice, chemotherapy regimens are chosen depending on the cancer type, tumour stage, patient's general state and preference.^11^ Most chemotherapy regimens are not highly emetic, and effective prophylaxis is available. Moreover, with an increasing number of molecular‐targeted and immunological therapies, more agents with low and minimal emetic potentials are becoming available.^12^

However, the side effects of chemotherapy remain a major concern of patients with cancer.^1^, ^13^ Some patients refuse chemotherapy^14^, ^15^ for fear of its side effects.^16^ Previous studies have reported a chemotherapy non‐compliance rate of 5%–18%.^14^, ^17^ In addition, avoidance of a treatment‐related decrease of their quality of life was the primary reason for refusal, and the fear of nausea before chemotherapy initiation was found to be a strong predictor of subsequent nausea.^18^ These data suggest that the patients' concern about treatment, which may be a result of their negative perception of high‐risk emetic agents and the lack of antiemetic therapy in early days, can deprive them from receiving appropriate treatment. This fear may be due to limited knowledge of CINV and chemotherapy‐specific anti‐emetic therapy.

Thus, awareness of the frequency of CINV and its management may help curb patients' negative perceptions of chemotherapy and ensure a more objective treatment decision‐making. A nation‐wide survey reported good compliance with the guidelines of anti‐emetic therapy^19^; however, the findings did not reflect those of a real‐world setting as the registry contained clinical information of patients from selected institutions, and a detailed analysis according to cancer type was not performed.

Using health utilisation data from the Hospital‐Based Cancer Registries (HBCR), this study aimed to describe the actual frequency of emetic chemotherapy use and the appropriate prophylaxis by cancer type.

## 2 METHODS

### 2.1 Study design and data source

This observational study used data from the Quality Indicator project,^20^ which involved health utilisation data linked with the HBCR. The project was conducted to evaluate the quality of health care of patients with cancer. In brief, the National Cancer Centre collects HBCR data from designated cancer care hospitals and voluntarily participating hospitals nationwide in Japan, covering approximately 70% of all patients with newly diagnosed cancer.^21^ Among these hospitals, 475 hospitals participated in the Quality Indicator project. We analysed health utilisation data collected from 1 January 2016 through 31 December 2017 for patients diagnosed with cancer in 2016. Collection of health utilisation data was part of the governmental survey that assesses the effect of the introduction of the diagnosis procedure combination‐based payment system. The survey data included information equivalent to fee‐for‐service insurance claims that cover all billable health services (e.g., diagnostic tests, imaging workup, procedures, treatments, and prescribed drugs) for both in‐ and out‐patients. These data were linked to the HBCR data of each patient in the participating hospitals. The data of approximately 49% of incident cancer cases in Japan were included in this study.

### 2.2 Patient selection

The subjects of this study were selected as follows. First, patients with cancer who were at least 20 years old at the time of diagnosis in 2016 and who were initiated on chemotherapy (intravenous and/or oral) were included in the study. Second, information on chemotherapeutic agents and antiemetic drugs were extracted from the utilisation data. Patients who received chemotherapy with interferon‐alpha were excluded as interferon‐alpha is used in other conditions like hepatitis C virus infection. For patients who received chemotherapy more than once, data of the first chemotherapy session after diagnosis was analysed. Many chemotherapeutic agents were administered within the first 8 days in one course (e.g., for patients with gastric cancer, cisplatin was added on Day 8 after S‐1 [tegafur/gimeracil/oteracil potassium] initiation). Thus, all drugs administered within the first 8 days were systematically included in one combination regimen in the emetic risk classification. Patients in whom chemotherapy was administered on the same day as surgery, thoracic drainage, abdominal drainage, and/or pericardial drainage were excluded because the chemotherapy drugs might have been administered topically. Patients who received chemotherapy drugs requiring arterial injection were excluded. Further, patients who received haematopoietic stem cell transplantation within 3 weeks of chemotherapy were also excluded because the emetic risks and prophylactic anti‐emetic drugs are different from those for regular chemotherapy.

### 2.3 Emetic risk classification

The emetic risk of chemotherapy was classified using the Japan Society of Clinical Oncology guidelines (JSCO).^22^ The guidelines of the National Comprehensive Cancer Network (NCCN),^6^ American Society of Clinical Oncology (ASCO),^23^ and Multinational Association Supportive Care in Cancer (MASCC) were used to classify the acute emetogenicity of chemotherapy drugs.^24^ The major differences between the guidelines are presented in Table 1. The emetic risks of some drugs differed according to dosage. Thus, the emetic risk of these drugs was based on the average Japanese adult's body surface area of 1.48 m^2^.^13^, ^25^ Finally, we classified cyclophosphamide administered at >1500 mg/m^2^ as HEC and methotrexate sodium administered at >250, 50–250, and <50 mg/m^2^ as HEC, MEC, and LEC, respectively.

**TABLE 1:** Major differences between the classifications of emetic risk of intravenous chemotherapy drugs

	Japan	ASCO	NCCN	MASCC/ESMO
High‐risk			Carboplatin (AUC ≥4)	
			Doxorubicin (≥60 mg)	
			Epirubicine (>90 mg)	
			Ifosfamide (≥2 g/m^2^ per dose)	
Moderate‐risk	Carboplatin	Carboplatin	Carboplatin (AUC < 4)	Carboplatin
	Epirubicine	Epirubicine		Epirubicine
	Ifosfamide	Ifosfamide		Ifosfamide
	Doxorubicin	Doxorubicin		Doxorubicin
	Cytarabine (>200 mg/m^2^)	Cytarabine (>1000 mg/m^2^)	Cytarabine (>200 mg/m^2^)	Cytarabine (>1000 mg/m^2^)
	Methotrexate (≥250 mg/m^2^)		Methotrexate (≥250 mg/m^2^)	
Low‐risk	Methotrexate (50–250 mg/m^2^)	Methotrexate	Methotrexate (50–250 mg/m2)	Methotrexate
		Bortexomib		Bortexomib
		Cetuximab		Cetuximab
		Nelarabine		
		Panitumumab		Panitumumab
		Pertuzumab		
Minimal‐risk	Bortexomib		Bortexomib	
	Cetuximab		Cetuximab	
	Nelarabine		Nelarabine	
	Panitumumab		Panitumumab	
	Pertuzumab		Pertuzumab	

*Note*: Japan: The 2015 Japan Society of Clinical Oncology Clinical Practice Guidelines for Antiemesis. ASCO: American Society of Clinical Oncology Antiemetics guidelines, *J Clin Oncol* 2020; 38:2782–2797. NCCN: National Comprehensive Cancer Network, Antiemesis guidelines, version 2.2020. MASCC: MASCC and ESMO consensus guidelines for the prevention of chemotherapy and radiotherapy‐induced nausea and vomiting: ESMO clinical practice guidelines. *Ann Oncol* 2016; 27:v119–v133.

Some of the emetic risks were defined based on the combination of the drugs.^22^ That is, fluorouracil, levoholinato, oxaliplatin, and irinotecan (FOLFOXIRI) for colorectal cancer and oxaliplatin, irinotecan, fluorouracil, and levoholinato (FOLFIRINOX) for pancreatic cancer were classified as HEC; gemcitabine and S1 (GS) and gemcitabine and nab‐paclitaxel (GEM/nab‐PTX) for pancreatic cancer as MEC; ifosfamide, carboplatin, and etoposide (ICE) for malignant lymphoma as HEC; and oral etoposide, nimustine, and ranimustine for malignant lymphoma as MEC.

### 2.4 Statistical analysis

The frequency of chemotherapy use by emetic risk was calculated in both the overall population and by cancer type. The prescription rate of the prophylactic antiemetic drugs according to the type of cancer and typical regimens in each cancer was also calculated. In general, antiemetic drugs administered on the same day as the first chemotherapy were regarded as prophylactic. For patients who received HEC after the initiation of chemotherapy with lower emetic risks (e.g., S1 + cisplatin is added on Day 8 for gastric cancer) or antiemetic drugs that were prescribed on the same day as HEC were considered prophylactic. To capture oral antiemetic drugs administered in preparation for the initiation of chemotherapy, oral drugs that were prescribed 30 days before chemotherapy initiation were also considered prophylactic. The disease stage was assessed by combining the clinical and pathological stages; the pathological stage was used for patients who underwent surgical resection, whereas the clinical stage was used for patients with unavailable data on the pathological stage of the tumour.^26^ All statistical analyses were performed using Stata software (ver. 15.0; Stata Corporation, Texas, USA).

## 3 RESULTS

In total, 172 133 patients receiving chemotherapy were identified (Table 2), among whom 70.4% received intravenous chemotherapy. The mean age of the study population was 65.9 (standard deviation, *SD* 12.0) years. The oral chemotherapy group was slightly older than the intravenous chemotherapy group (68.7 vs. 64.1 years). Non‐small cell lung cancer was the most common cancer type (14.1%), followed by colorectal cancer (12.9%) and breast cancer (9.9%). A larger proportion of patients who received oral chemotherapy had gastric and colorectal cancers (57.7% and 55.3%, respectively) compared with other cancers (4.7%–36.8%). A total of 62.5% of prescribed oral chemotherapy was adjuvant chemotherapy.

**TABLE 2:** Patients' characteristics

	Intravenous chemotherapy group		Oral chemotherapy group		Total	
	*N*121 103	(%)100.0	*N*51 030	(%)100.0	*N*172 133	(%)100.0
Sex, male	64 079	52.9	30 084	59.0	94 163	54.7
Age (years), mean (*SD*)	64.7	(SD: 12.2)	68.7	(SD: 11.2)	65.9	(SD: 12.0)
Cancer type						
Non‐small cell lung	15 991	13.2	8264	16.2	24 255	14.1
Colorectal	9932	8.2	12 265	24.0	22 197	12.9
Breast	15 861	13.1	1262	2.5	17 123	9.9
Gastric	6772	5.6	9246	18.1	16 018	9.3
Malignant lymphoma	13 190	10.9	643	1.3	13 833	8.0
Pancreatic	6946	5.7	4039	7.9	10 985	6.4
Oesophageal	6922	5.7	516	1.0	7438	4.3
Small cell lung cancer	4211	3.5	37	0.1	4248	2.5
Others	41 278	34.1	14 758	28.9	56 036	32.6
Stage						
Stage 0	2724	2.2	859	1.7	3583	2.1
Stage I	16 717	13.8	7870	15.4	24 587	14.3
Stage II	20 658	17.1	10 687	20.9	31 345	18.2
Stage III	29 704	24.5	12 662	24.8	42 366	24.6
Stage IV	41 576	34.3	12 222	24.0	53 798	31.3
Unknown	9724	8.0	6730	13.2	16 454	9.6
Adjuvant chemotherapy	51 148	42.2	31 873	62.5	83 021	48.2

Abbreviation: SD, standard deviation.

**FIGURE 1:**
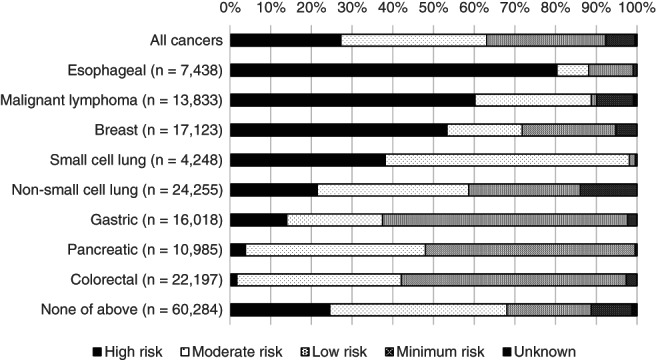
Distribution of chemotherapy emetic risk by cancer type

The most prescribed chemotherapy was MEC (*n* = 60 528, 35.2%), followed by LEC (*n* = 51 645, 30.0%), and HEC (*n* = 46 458, 27.0%; Table 3 and Figure 1). In the intravenous chemotherapy group, 47.0% (*n* = 56 911) and 38.2% (*n* = 46 306) received MEC and HEC, respectively. Further, more than 50% of the patients who received HEC were prescribed cisplatin (*n* = 27 933). Meanwhile, 40.7% (*n* = 23 171) of the patients who received MEC were administered carboplatin. In the oral chemotherapy group, 39 446 (77.3%) patients received LEC, and approximately 50% of them (*n* = 18 740) received S1.

**TABLE 3:** Distribution of emetic risk by antineoplastic agent and mode of administration

	Intravenous chemotherapy		Oral chemotherapy		Total	
	*N*121 103	(%)100.0	*N*51 030	(%)100.0	*N*172 133	(%)100.0
High emetic risk	46 306	38.2	152	0.3	46 458	27.0
Cisplatin (IV)	27 933	60.3	–		27 933	60.1
Doxorubicin and cyclophosphamide (IV)	10 143	21.9	–		10 143	21.8
Epirubicine and cyclophosphamide (IV)	6925	15.0	–		6925	14.9
Procarbazine (PO)	–		152	100.0	152	0.3
Moderate emetic risk	56 911	47.0	3617	7.1	60 528	35.2
Carboplatin (IV)	23 171	40.7	–		23 171	38.3
Oxaliplatin (IV)	12 145	21.3	–		12 145	20.1
Cyclophosphamide (IV) ≤1500 mg	6251	11.0	–		6251	10.3
Temozolomide (PO)	–		1566	43.3	1566	2.6
Imatinib (PO)	–		960	26.5	960	1.6
Cyclophosphamide (PO)	–		606	16.8	606	1.0
Low emetic risk	12 199	10.1	39 446	77.3	51 645	30.0
Gemcitabine (IV)	3146	25.8	–		3146	6.1
Docetaxel (IV)	3360	27.5	–		3360	6.5
Paclitaxel (IV)	3006	24.6	–		3006	5.8
Mitomycin C (IV)	1064	8.7	–		1064	2.1
Fluorouracil(IV)	667	5.5	–		667	1.3
S1 (tegafur/gimeracil/oteracil potassium) (PO)	–		18 740	47.5	18 740	36.3
UFT (tegafur/uracil) (PO)	–		9532	24.2	9532	18.5
Capecitabine (PO)	–		5328	13.5	5328	10.3
Lenalidomide hydrate (PO)	–		1036	2.6	1036	2.0
Afatinib maleate (PO)	–		974	2.5	974	1.9
Dasatinib hydrate (PO)	–		867	2.2	867	1.7
Sunitinib malate (PO)	–		770	2.0	770	1.5
Minimal emetic risk	4789	4.0	7786	15.3	12 575	7.3
Bortezomib (IV)	1482	30.9	–		1482	11.8
Rituximab (IV)	1230	25.7	–		1230	9.8
Trastuzumab (IV)	717	15.0	–		717	5.7
Gefitinib (PO)	–		1798	23.1	1798	14.3
Methotrexate (PO)	–		1672	21.5	1672	13.3
Hydroxycarbamide (PO)	–		1576	20.2	1576	12.5
Sorafenib (PO)	–		1375	17.7	1375	10.9
Erlotinib (PO)	–		946	12.2	946	7.5
Unknown	898	0.7	29	0.1	927	0.5

Abbreviations: iv, intravenous chemotherapy; po, oral chemotherapy.

The distribution of the chemotherapy emetic risk differed with cancer type. Table 4 shows the major chemotherapy regimens. HEC was commonly used for oesophageal cancer (80.3%), malignant lymphoma (60.2%), and breast cancer (53.8%). Among the patients with oesophageal cancer who received HEC, 77.5% received 5‐fluorouracil plus cisplatin (FP). Among patients with malignant lymphoma who were administered HEC, 84.1% received CHOP (cyclophosphamide, doxorubicin hydrochloride, vincristine, and prednisolone) with or without rituximab. MEC was used for small cell lung (59.9%), pancreatic (44.2%), and colorectal cancers (40.4%). Meanwhile, LEC was administered most in patients with gastric (60.2%), colorectal (55.2%), and pancreatic (51.6%) cancers.

**TABLE 4:** Examples of chemotherapy regimens and proportion of prescription for each emetic risk

Cancer type	Chemotherapy regimen			
	High risk	Moderate risk	Low risk	Minimal risk
Non‐small cell lung	CDDP + pemetrexed (35.3%) NP (35.6%)	TC* (48.8%)	UFT (53.0%) Pemetrexed (38.6%)	Gefitinib (49.6%) Erlotinib (30.4%)
Colorectal	FOLFOXIRI (30.2%)	CAPOX (58.7%) FOLFOX (22.1%)	Capecitabine (38.5%) UFT + LV (36.5%)	Panitumumab (17.9%) Regorafenib (14.9%)
Breast	FEC (43.1%) EC (31.9%) AC (24.6%)	TC** (85.8%)	PTX (35.0%) Trastuzumab + PTX (17.0%) DTX (12.9%)	Trastuzumab (74.7%)
Gastric	S‐1 + CDDP (64.4%) Cape + CDDP (17.7%)	SOX (68.4%)	S‐1 (88.7%)	Nivolumab (3.9%) Ramucirumab (3.6%)
Malignant lymphoma	CHOP (84.1%)	CPM <1500 mg (65.1%)	–	–
Pancreatic	FOLFIRINOX (79.8%)	GEM + nab‐PTX (91.3%)	S‐1 (69.6%) GEM (29.2%)	–
Oesophageal	FP (77.5%) DCF (17.6%)	Nedaplatin + 5‐FU (63.8%)	DTX (12.4%)	–
Small cell lung	PE (69.0%) PI (30.3%)	CBDCA + etoposide (88.8%) CBDCA+CPT11 (5.8%)	CPT11 (41.3%)	‐

*Note*: This table shows the percentage of the tumours' major regimens at each emetic risk. NP, CDDP + VNR; TC*, CBDCA + PTX; FOLFOXIRI, 5‐FU + l‐LV + L‐OHP + CPT11; CAPOX, capecitabine + L‐OHP; FOLFOX, 5‐FU + l‐LV + L‐OHP; FEC, 5‐FU + EPI + CPA; EC, EPI + CPA; AC, ADM + CPA; TC**, DTX + CPA; SOX, S‐1 + L‐OHP; CHOP, CPA + ADM + VCR + PSL; FOLFIRINOX, L‐OHP + CPT‐11 + 5‐FU + l‐LV; FP, 5‐FU + CDDP; DCF, DTX + CDDP + 5‐FU; PE, CDDP + etoposide; PI, CDDP + CPT11.

Abbreviations: 5‐FU, 5‐fluorouracil; ADM, doxorubicin hydrochloride; CBDCA, carboplatin; CDDP, cisplatin; CPA, cyclophosphamide; CPM, cyclophosphamide; CPT11, irinotecan hydrochloride hydrate; DTX, docetaxel hydrate; EPI, epirubicine hydrochloride; GEM, gemcitabine hydrochloride; l‐LV, levofolinate calcium; L‐OHP, oxaliplatin; LV, folinate; PSL, prednisolone; PTX, paclitaxel; S‐1, tegafur/gimeracil/oteracil potassium; UFT, tegafur/uracil; VCR, vincristine sulphate; VNR, vinorelbine ditartrate.

**FIGURE 2:**
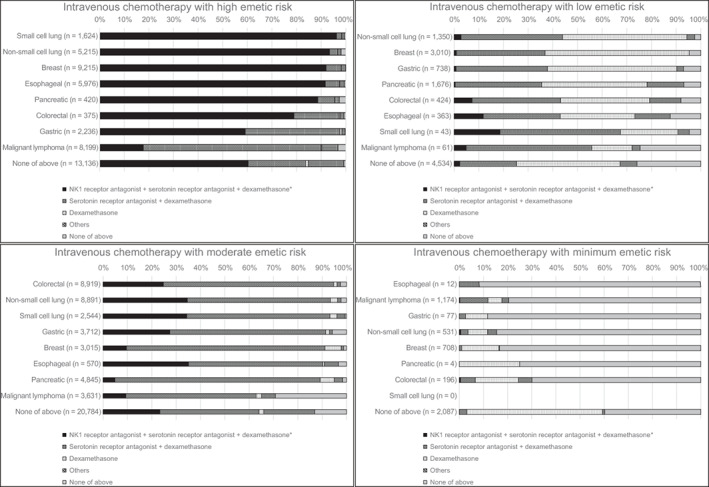
Distribution of prescription of antiemetic drugs by cancer type

Table 5 shows the prescription rate of the prophylactic antiemetic drugs. In the intravenous chemotherapy with HEC subgroup, 70.7% (95% confidence interval [CI], 70.3%–71.1%) of the patients were prescribed a three‐drug combination comprising an NK_1_ receptor antagonist, a serotonin receptor antagonist, and dexamethasone. In the intravenous chemotherapy with MEC subgroup, 59.1% (95% CI, 58.7%–59.5%) were prescribed a two‐drug combination, and 24.0% (95% CI, 23.7–24.4) were prescribed a three‐drug combination. Among the patients who received HEC, the prescription rates of antiemetic drugs differed with cancer type (Figure 2). Among the patients with small cell lung, non‐small cell lung, breast, and oesophageal cancers who were administered intravenous chemotherapy with HEC, 96.2% (95% CI, 95.1–97.1), 93.4% (95% CI, 92.7–94.1), 92.9% (95% CI, 92.4–93.4), and 91.6% (95% CI, 90.9–92.3) were prescribed a three‐drug antiemetic regimen, respectively. Meanwhile, 17.7% (95% CI, 16.8–18.5) of the patients with malignant lymphoma treated with HEC were prescribed this regimen. Antiemetic therapy according to chemotherapy regimen for each cancer type was shown in Appendix S1. More than 90% of patients who received HEC regimen were prescribed the recommended three‐drug antiemetic regimen, while 13.5% of patients with malignant lymphoma receiving CHOP were prescribed these antiemetic regimen.

**TABLE 5:** Prescription of antiemetic drugs

	Intravenous chemotherapy (*n* = 121 103)	Oral chemotherapy (*n* = 51 030)	Total (*n* = 172 133)
	%, CI	%, CI	%, CI
High emetic risk			
NK1 receptor antagonist + serotonin receptor antagonist + dexamethasone^a^	70.7 (70.3–71.1)	4.6 (1.9–9.3)	70.5 (70.1–70.9)
Serotonin receptor antagonist + dexamethasone^a^	24.7 (24.3–25.1)	34.9 (27.3–43.0)	24.7 (24.3–25.1)
Serotonin receptor antagonist	1.8 (1.7–2.0)	10.5 (6.1–16.5)	1.8 (1.7–2.0)
Dexamethasone	0.5 (0.4–0.6)	16.4 (10.9–23.3)	0.6 (0.5–0.6)
None of above	1.2 (1.1–1.3)	32.9 (25.5–41.0)	1.3 (1.2–1.4)
Moderate emetic risk			
NK1 receptor antagonist + serotonin receptor antagonist + dexamethasone^a^	24.0 (23.7–24.4)	0.0 (0.0–0.2)	22.6 (22.3–22.9)
Serotonin receptor antagonist + dexamethasone^a^	59.1 (58.7–59.5)	10.6 (9.6–11.6)	56.2 (55.8–56.6)
Serotonin receptor antagonist	3.7 (3.6–3.9)	13.7 (12.6–14.9)	4.3 (4.2–4.5)
Dexamethasone	2.7 (2.6–2.8)	10.6 (9.6–11.7)	3.2 (3.0–3.3)
None of above	8.6 (8.4–8.8)	64.6 (63.0–66.1)	11.9 (11.7–12.2)
Low emetic risk			
NK1 receptor antagonist + serotonin receptor antagonist + dexamethasone^a^	2.1 (1.9–2.4)	0.4 (0.3–0.5)	0.8 (0.8–0.9)
Serotonin receptor antagonist + dexamethasone^a^	31.6 (30.8–32.4)	0.8 (0.7–0.8)	8.0 (7.8–8.3)
Serotonin receptor antagonist	4.7 (4.3–5.0)	0.6 (0.5–0.7)	1.5 (1.4–1.7)
Dexamethasone	46.9 (46.0–47.8)	3.6 (3.4–3.8)	13.8 (13.5–14.1)
None of above	13.3 (12.7–13.9)	93.8 (93.6–94.1)	74.8 (74.4–75.2)
Minimum emetic risk			
NK1 receptor antagonist + serotonin receptor antagonist + dexamethasone^a^	0.2 (0.0–0.3)	0.0 (0.0–0.0)	0.0 (0.0–0.1)
Serotonin receptor antagonist + dexamethasone^a^	5.0 (4.4–5.6)	0.1 (0.0–0.2)	2.0 (1.7–2.2)
Serotonin receptor antagonist	0.9 (0.7–1.2)	0.2 (0.1–0.3)	0.5 (0.4–0.6)
Dexamethasone	30.0 (28.7–31.3)	2.6 (2.3–3.0)	13.0 (12.4–13.6)
None of above	63.1 (61.7–64.5)	96.8 (96.3–97.1)	83.9 (83.3–84.6)

*Note*: None: No prescription of an NK1 receptor antagonist, serotonin receptor antagonist, and dexamethasone.

^a^ Any steroid included.

In the oral chemotherapy group, the two‐drug regimen was prescribed for 34.9% (95% CI, 27.3–43.0) and 10.6% (95% CI, 9.6–11.6) of the patients treated with HEC and MEC, respectively. The rate of use of a single serotonin receptor antagonist for HEC and MEC was 10.5% and 13.7%, respectively.

## 4 DISCUSSION

This study showed that MEC was used more than HEC, and CINV prophylaxis was widely used for HEC in a real‐world setting in Japan. The rate of prescription of HEC was dependent on the cancer type and was highest for patients with oesophageal cancer (80.3%), malignant lymphoma (60.2%), and breast cancer (53.8%). Meanwhile, MEC was prescribed mostly for patients with small cell lung (59.9%), pancreatic (44.2%), and colorectal (40.4%) cancers. For LEC, it was administered mostly for patients with gastric, colorectal, and pancreatic cancers, accounting for 50% of the patients who received chemotherapy. Further, the guidelines for antiemetic prophylaxis were used widely in 2016; meanwhile, some guidelines were revised in 2016.

Patients with cancer and an indication for chemotherapy often experience pre‐treatment psychological distress.^27^ A previous study showed that pre‐chemotherapy education can decrease treatment‐related concerns and improve physical/psychological outcomes.^28^ Therefore, psychoeducational support can be an effective intervention for managing CINV.^29^, ^30^ To avoid treatment refusal due to strong concerns about CINV, health care providers should establish a system to educate patients on the CINV risk of their planned chemotherapy regimen. Further, it should be emphasised that chemotherapy should be accompanied by a recommended anti‐emetic therapy. Moreover, the actual risk may depend on the patients' characteristics (cancer and treatment type, patient's age and sex),^31^ and thus these factors may need to be incorporated in the educational materials.

A nationwide survey reported a good compliance to the guidelines for anti‐emetic therapy.^19^ Using the health utilisation data linked with the HBCR, this study described the actual frequency of using emetic chemotherapy and the appropriate prophylaxis by cancer type and typical regimens. The overall adherence to prophylactic antiemetic drugs for intravenous chemotherapy in this study was higher than that in previous studies in Japan.^32^, ^33^ However, this study found that only 13.5% of patients with malignant lymphoma treated with CHOP received the recommended antiemetic therapy; this was consistent with a previous study.^34^ CHOP therapy use high dose prednisolone administration. Therefore, it may be recognised in clinical practice that many patients receiving CHOP do not suffer CINV. Aapro et al. reported that guideline‐consistent antiemetic therapy alleviates CINV significantly.^5^ Healthcare professionals should consider using recommendations from guidelines.

Assuming that the effectiveness of the antiemetic drugs was similar to that in previous reports (i.e., in patients receiving both intravenous HEC and the recommended antiemetic, the frequency of vomiting was 30%) and that 90% of the patients received the anti‐emetic prophylaxis for HEC, the frequency of vomiting in the patients who received HEC without antiemetic prophylaxis was approximately twice that of the patients who took HEC with prophylaxis. Furthermore, considering that more than 90% of patients with non‐small cell lung, breast, and oesophageal cancers who received HEC with the recommended antiemetic treatment in 2016, the frequency of vomiting was approximately 35%. Education of patients on the risk of CINV may reduce excessive concerns about CINV.

The appropriateness of antiemetic prophylaxis for oral chemotherapy could not be evaluated because the recommendations for prophylactic antiemetic drugs for oral chemotherapy vary with existing guidelines because of limited information on the emetic risk of oral chemotherapeutic agents.^6^, ^35^, ^36^ For example, the NCCN guideline recommend the use of a single serotonin receptor antagonist for patients with a high‐to‐moderate risk of CINV.^8^ Meanwhile, the MASCC guideline recommends a two‐drug combination of a serotonin receptor antagonist and dexamethasone for the same patients.^37^ This study simply describes the current status of prophylactic antiemetic drugs prescribed for patients receiving oral chemotherapy. These findings may be used for further research on the appropriate antiemetic therapy for these patients.

This study had some limitations. First, this study did not measure the frequency of CINV due to unavailability of the CINV incidence data. Therefore, we estimated the frequency using data from a previous study. Second, antiemetic prophylaxis was defined based on the time of prescription recorded in the database. Although prophylactic antiemetic drugs prescribed on the same day with chemotherapy or within 30 days of oral chemotherapy were most likely prophylactic, the possibility that they were actually prescribed for therapeutic purposes could not be excluded. Third, this study examined the situation of patients diagnosed in 2016. The NCCN and ASCO antiemetic guidelines have been revised after 2016. The new guidelines also recommended additional use of olanzapine for HEC.^6^, ^23^ We did not examine this recommendation. Fourth, the authors did not consider the patients' individual risk factors for CINV, such as sex, morning sickness, and comorbidities. Incorporating these factors may justify the non‐prescription of antiemetic prophylaxis in patients receiving HEC. Finally, the exact drug dosages per the body surface area of the patients were not available, because information on the patients' body surface area was not available in the claims data. Consequently, the emetic risks of patients receiving chemotherapy with agents, such as methotrexate and cytarabine, may be misclassified.^33^ Despite these limitations, these findings will be helpful in understanding the real‐world clinical situation of the CINV risk and prophylactic antiemetic use in Japan. Further studies should evaluate the effect of prophylactic education with/without psychoeducational support on relieving patients' concerns.

## 5 CONCLUSION

Overall, HEC was less prescribed than MEC, and prophylactic antiemetic drugs were generally prescribed. Healthcare professionals should educate patients about emetic risks before chemotherapy initiation to avoid patients' concerns about CINV that may lead to treatment termination. This type of survey should be repeated to observe the improvements in compliance with recommended anti‐emetic therapy.

## CONFLICT OF INTEREST

Narikazu Boku received funds from Ono and Takeda, and honorarium from Ono and Taiho. All the other authors declare no conflict of interest.

## AUTHOR CONTRIBUTIONS

All authors had full access to the data in the study and take responsibility for the integrity of the data and the accuracy of the data analysis. *Conceptualization*, A.O., T.H.; *Methodology*, A.O.; *Investigation*, A.O.; *Formal Analysis*, A.O; *Writing—Original Draft*, A.O; *Writing—Review & Editing*, A.O., N.B., T.H.; *Supervision*, N.B., T.H.; *Founding*, A.O.; *Data Collection*, T.H.

## ETHICS STATEMENT

This study was approved by the institutional review board of the National Cancer Centre in Japan (2018–270) and was conducted according to the Declaration of Helsinki.

## Supporting information


**Appendix** **S1.** Supporting Information.

## Data Availability

Our data was permitted for use for only this research.
