# Supplementary figures and images for: Real‐world emetic risk of chemotherapy and the corresponding antiemetic therapy in Japan: A study based on a nationwide database
Source: Cancer Rep (Hoboken). 2021 Jun 27;5(3):e1482. doi: 10.1002/cnr2.1482 (PMC8955058; doi:10.1002/cnr2.1482)

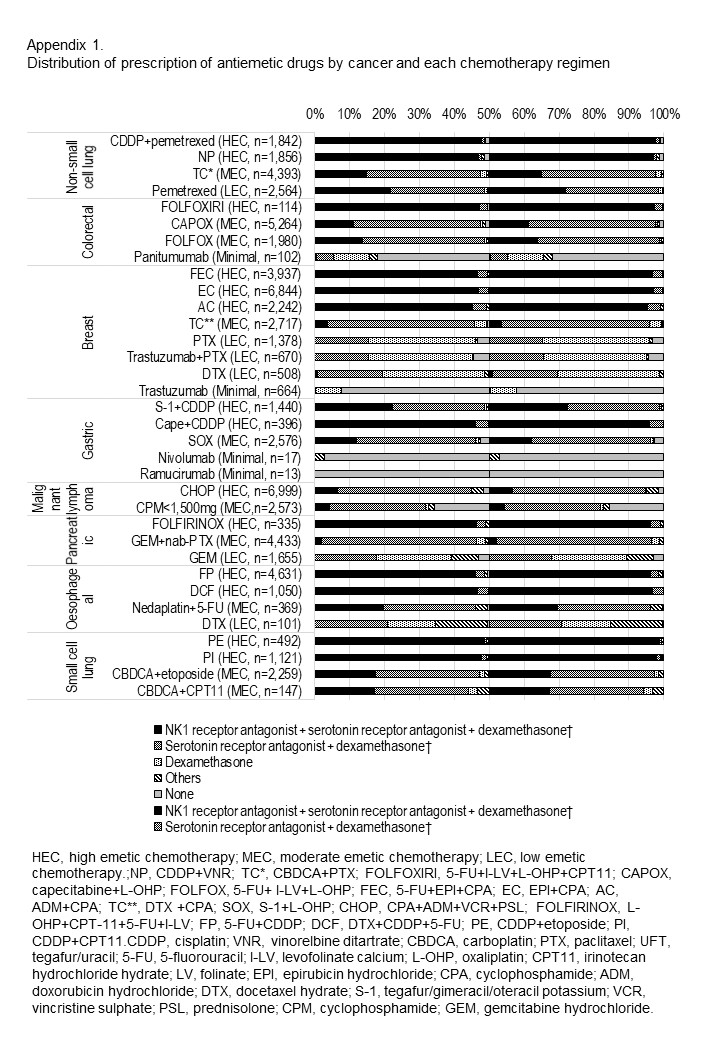

Supplement: Supplementary file 1 — Appendix S1. Supporting Information. [file CNR2-5-e1482-s001.jpg]
